# Pancreas-specific activation of mTOR and loss of p53 induce tumors reminiscent of acinar cell carcinoma

**DOI:** 10.1186/s12943-015-0483-1

**Published:** 2015-12-18

**Authors:** Bo Kong, Tao Cheng, Chengjia Qian, Weiwei Wu, Katja Steiger, Jing Cao, Anna Melissa Schlitter, Ivonne Regel, Susanne Raulefs, Helmut Friess, Mert Erkan, Irene Esposito, Jörg Kleeff, Christoph W. Michalski

**Affiliations:** Department of Surgery, Technische Universität München (TUM), Munich, Germany; Institute of Pathology, TUM, Munich, Germany; Department of Surgery, Koc School of Medicine, Istanbul, Turkey; Institute of Pathology, Heinrich-Heine-Universität Düsseldorf, Düsseldorf, Germany; Royal Liverpool and Broadgreen University Hospitals, Liverpool, UK; Department of Surgery, University of Heidelberg, Im Neuenheimer Feld 110, 69120 Heidelberg, Germany

**Keywords:** Pancreatic cancer, p53, mTOR, ACC, Organ involution, Tissue homeostasis

## Abstract

**Background:**

Pancreatic acinar cell carcinoma (ACC) is a rare tumor entity with an unfavorable prognosis. Recent whole-exome sequencing identified p53 mutations in a subset of human ACC. Activation of the mammalian target of rapamycin (mTOR) pathway is associated with various pancreatic neoplasms. We thus aimed at analyzing whether activation of mTOR with a concomitant loss of p53 may initiate ACC.

**Methods:**

We generated transgenic mouse models in which mTOR was hyperactivated through pancreas-specific, homozygous *tuberous sclerosis 1* (*Tsc1*) deficiency, with or without deletion of p53 (*Tsc1*^*-/-*^ and *Tsc1*^*-/-*^*; p53*^*-/-*^). Activity of mTOR signaling was investigated using mouse tissues and isolated murine cell lines. Human ACC specimens were used to corroborate the findings from the transgenic mouse models.

**Results:**

Hyperactive mTOR signaling in *Tsc1*^*-/-*^ mice was not oncogenic but rather induced a near-complete loss of the pancreatic acinar compartment. Acinar cells were lost as a result of apoptosis which was associated with p53 activation. Concomitantly, ductal cells were enriched. Ablation of p53 in *Tsc1*-deficient mice prevented acinar cell death but promoted formation of acinar cells with severe nuclear abnormalities. One out of seven *Tsc1*^*-/-*^*; p53*^*-/-*^ animals developed pancreatic tumors showing a distinctive tumor morphology, reminiscent of human ACC. Hyperactive mTOR signaling was also detected in a subset of human ACC.

**Conclusion:**

Hyperactive mTOR signaling combined with loss of p53 in mice induces tumors similar to human ACC.

**Electronic supplementary material:**

The online version of this article (doi:10.1186/s12943-015-0483-1) contains supplementary material, which is available to authorized users.

## Background

Pancreatic acinar cell carcinoma (ACC) is a rare exocrine cancer which accounts for 1–2 % of pancreatic malignancies [1, 2]. On histological and molecular levels, ACCs display certain features of normal pancreatic acini by expressing exocrine enzymes (e.g., trypsin) and by forming small glandular structures [3]. In comparison to pancreatic ductal adenocarcinoma (PDAC), ACCs tend to have a relatively favorable tumor biology which is more likely to respond to the available therapies [4–7]. Historically, it is conceived that ACCs do not share typical mutations of PDAC such as p53 [3, 8]. However, this view has recently been updated by whole-exome sequencing of ACCs [9]. Here, 13 % of ACC samples contained somatic p53 mutations and 39 % of them had a loss of heterozygosity at the p53 locus [9]. Therefore, it is likely that the p53 pathway actively participates in the development of a subset of ACCs.

The mammalian target of rapamycin (mTOR) is an atypical serine/threonine kinase that interacts with other proteins to form two functional complexes: mTORC1 and mTORC2 [10]. Particularly, mTORC1 (referred to hereinafter as mTOR) integrates a variety of intracellular and extracellular cues and is a key regulator of protein synthesis. It potentially influences carcinogenesis at various stages including cancer initiation and metastasis [11, 12]. The tuberous sclerosis 1 and 2 complex (Tsc1-Tsc2 complex) negatively regulates mTOR activity [13–16].

There is increasing evidence that mTOR activation is a common event in the development of various pancreatic tumors such as PDAC, a subset of cystic tumors and pancreatic neuroendocrine tumors (PNETs, [17–19]). Furthermore, we have previously demonstrated that the oncogenic effect of mTOR largely relies on its activation pattern: PI3K/Akt (phosphatidylinositol-4, 5-bisphosphate 3-kinase/protein kinase B)-mediated mTOR signaling induced cystic pancreatic lesions whereas oncogenic Mek/Erk-induced mTOR signaling promoted metastatic PDACs [18]. Since p53 interacts with mTOR signaling at multiple levels via the Tsc1-Tsc2 complex [20, 21], we aimed to analyze whether hyperactivated mTOR signaling with a loss of p53 may specify ACC.

## Results

### Tsc1 deficiency triggers loss of acinar cells in the exocrine pancreas

Previously, we generated pancreas-specific *Tsc1*-haploinsufficient (*p48*^*Cre/+*^; *Tsc1*^*fl/+*^) mice by crossing the *Ptf1a*^*Cre/+*^ line (also known as *p48*^*Cre/+*^) with the *Tsc1*^*fl/fl*^ line in which one allele of *Tsc1* is specifically ablated in pancreatic progenitors during embryonic development and continuously deleted in pancreatic acinar cells in adulthood. However, neither overt pathologies nor hyperactive mTOR signaling was observed in the pancreata of these animals [18]. To further investigate the role of mTOR signaling in pancreatic exocrine physiology, we thus generated transgenic *p48*^*Cre/+*^*; Tsc1*^*fl/fl*^ mice (herein referred to as *Tsc1*^*-/-*^). A complete loss of *Tsc1* expression in the pancreata of these transgenic animals was previously confirmed [18]. The *p48*^*Cre/+*^; *Tsc1*^*fl/+*^ mice (herein referred to as *Tsc1*^*+/-*^) were used as controls.

**Fig. 1:**
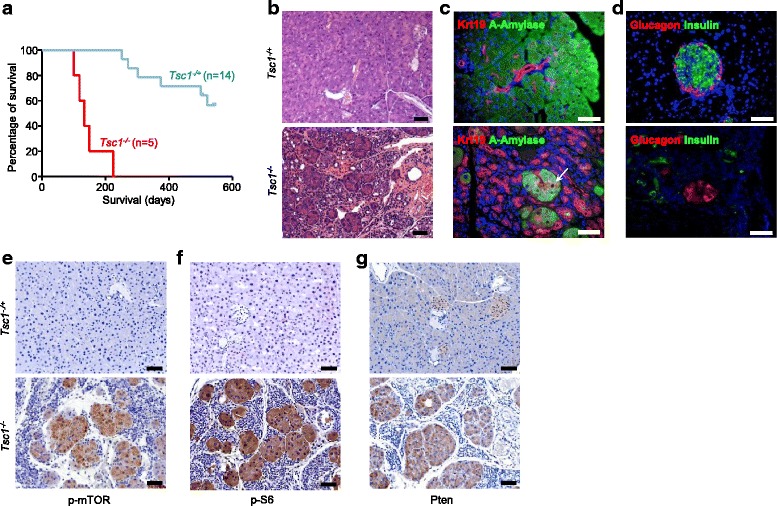
*Tsc1* deficiency triggers loss of acinar cells in the exocrine pancreas. (**a**) Kaplan–Meier survival analysis shows *Tsc1* ^*-/-*^ mice survival time (median survival: 131 days; *n* = 5); (**b**), Representative H&E-stained sections of *Tsc1* ^*-/+*^ and *Tsc1* ^*-/-*^ pancreata show significant loss parenchymal cells in *Tsc1* ^*-/ -*^mice, but not in *Tsc1* ^*-/+*^ mice; scale bar: 50 μm (**c**-**d**), Co-IF for Krt19, α-amylase, glucagon and insulin show the loss of tissue homeostasis in the endocrine and exocrine compartment of *Tsc1* ^*-/-*^ pancreata; scale bars: 50 μm; (**e**-**g**), Representative IHC pictures show distinct in vivo activation of mTOR signaling (p-mTOR^Ser2448^ and p-S6^Ser235/236^) and induction of Pten in pancreatic tissues from *Tsc1* ^*-/-*^ mice, scale bar: 50 μm

All *Tsc1*^*-/*-^ mice died between the age of 100 days and 224 days (*n* = 5; median survival: 133 days; Fig. 1a). Histological examination of *Tsc1*^*-/-*^ pancreata revealed a destruction of the normal pancreatic acinar cell compartment (50–80 %) with a relative enrichment of ductal cells. However, no such changes were observed in *Tsc1*^*-/+*^ (*n* = 14) pancreata (Fig. 1b). The number of acinar cells was reduced and the residual acinar cells were surrounded by ductal cells in *Tsc1*^*-/-*^ pancreata (α-amylase and Krt19 double-IF; Fig. 1c). Similar to previous observations in *Tsc2*-deficient mice [22], hyperglycemic episodes were observed in two *Tsc1*^*-/-*^ mice by the time of death/sacrifice and the number of β cells was also significantly reduced (blood glucose levels of 370 and 590 mg/dl, respectively; normal: 160 to 200 mg/dl, insulin and glucagon double-IF; Fig. 1d). These data suggest that the *Tsc1*^*-/-*^ mice suffered from both exocrine and endocrine insuffiency by the time of sacrifice. However, the *Tsc1*^*-/+*^ pancreata were histologically normal. Taken together, *Tsc1* deficiency caused loss of functional parenchymal cells in both the exocrine and endocrine compartment of the pancreas. However, *Tsc1* haploinsufficiency did not result in a gross pathological phenotype.

Next, we aimed to determine whether *Tsc1* deficiency resulted in hyperactive mTOR signaling in the pancreas. IHC studies revealed that residual acinar cells in *Tsc1*^*-/-*^ animals were strongly positive for p-mTOR^Ser2448^ and p-S6^Ser235/236^ while the acinar cells in *Tsc1*^*-/+*^ mice were only weakly positive (Fig. 1e, f). As a feedback response to hyperactive mTOR signaling, Pten was induced in residual acinar cells (Fig. 1g). No such differences were observed in *Tsc1*^*-/+*^ pancreata.

### Hyper-activated mTOR signaling induces p53 and apoptosis of acinar cells

**Fig. 2:**
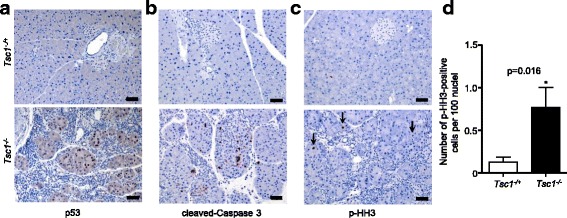
Hyperactivated mTOR signaling induces p53 and apoptosis in acinar cells. (**a**-**c**) Representative IHC pictures show in vivo activation of p53, apoptosis (cleaved-caspase 3), increased proliferation (p-Histone H3 (p-HH3)) in pancreatic tissues from *Tsc1* ^*-/-*^ mice, scale bar: 50 μm; (**d**) Quantification of p-HH3-positive cells in *Tsc1* ^*-/-*^ (*n* = 4) and *Tsc1* ^*-/+*^ (*n* = 4) pancreata; *: *p* < 0.05

We then set out to investigate potential reasons for the loss of acinar cells induced by hyper-activated mTOR signaling. Previous studies in mouse embryonic fibroblasts (MEFs) showed that hyper-activated mTOR rendered these cells susceptible to p53-dependent apoptosis [23]. Thus, expression of cleaved-caspase 3 (a marker of apoptosis) and p53 was analyzed, revealing that the residual acinar cells in *Tsc1*^*-/ -*^ pancreata were apoptotic and were strongly positive for nuclear p53 (Fig. 2a, b). In addition, an increased proliferation rate was observed in these residual acinar cells (Fig. 2c, d). Such changes were not seen in *Tsc1*^*-/+*^ pancreata; Fig. 2a, b and c).

### Loss of p53 and Tsc1 promote acinar cell transformation

**Fig. 3:**
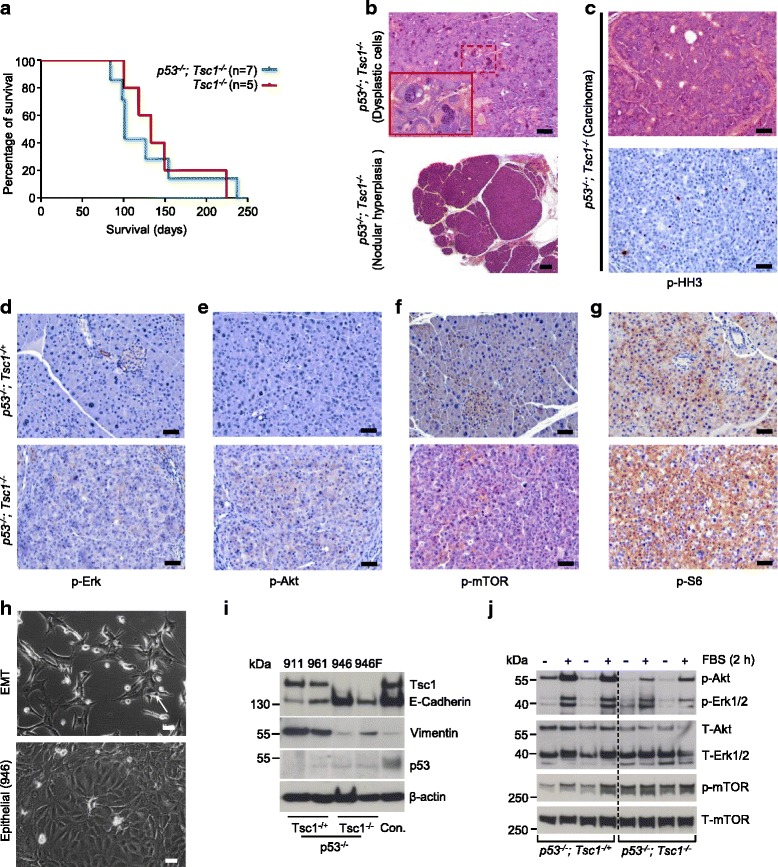
Loss of p53 and Tsc1 promote acinar cell transformation. (**a**) Kaplan–Meier survival analysis shows *p53* ^*-/-*^; *Tsc1* ^*-/-*^ mice survival time (median survival: 101 days; *n* = 7) which is not different from that of *Tsc1* ^*-/-*^ mice (median survival: 133 days; *n* = 5); (**b**) Representative H&E-stained sections of *p53* ^*-/-*^; *Tsc1* ^*-/+*^ and *p53* ^*-/-*^; *Tsc1* ^*-/-*^ pancreata show pancreatic acinar cells with nuclear abnormalities (upper panel) and nodular hyperplasia (lower panel); scale bar: 50 μm (upper panel) and 200 μm (lower panel); (**c**) Representative H&E-stained sections and IHC pictures show the histology of one ACC-like tumor with a high proliferative index (p-HH3); (**d**-**g**) Representative IHC pictures show distinct in vivo activation of mTOR signaling (p-mTOR^Ser2448^ and p-S6^Ser235/236^), but inactivation of Erk (p-Erk1/2^Thr202/Tyr204^) and Akt (p-Akt^Ser473^) in tumor cells (and atypical acinar cells) of pancreatic tissues from *p53* ^*-/-*^; *Tsc1* ^*-/-*^ mice, scale bar: 50 μm; (**h**) Phase contrast images of isolated cell lines from *p53* ^*-/-*^ *; Tsc1* ^*-/+*^ (911 and 961) and *p53* ^*-/-*^ *; Tsc1* ^*-/-*^ (946 and 946 F) mice show fibroblast-like (961, 911 and 946 F) and epithelial (946) morphologies, scale bar: 50 μm; (**i**) Western-blot analysis demonstrates expression of Tsc1, E-Cadherin, Vimentin and p53 in *p53* ^*-/-*^ *; Tsc1* ^*-/+*^ and *p53* ^*-/-*^ *; Tsc1* ^*-/-*^ cells; previously described cystic cells lines isolated from *P48* ^*Cre/+*^ *; Pten* ^*-/-*^ *; Tsc1* ^*-/+*^ mice were used as used as external controls [18]; (**j**) Western-blot analysis demonstrates phosphorylation levels of p-Akt^Ser473^, p-Erk1/2^Thr202/Tyr204^ and p-mTOR^Ser2448^ in p53^*-/-*^ *; Tsc1* ^*-/+*^ and *p53* ^*-/-*^ *; Tsc1* ^*-/-*^ cells after fetal bovine serum (FBS) treatment for 2 h; one of the three independent experiments is shown

To confirm that acinar cell apoptosis was p53-dependent, we inactivated p53 in the context of *Tsc1* deficiency. Thus, *p48*^*Cre/+*^*; p53*^*fl/fl*^*; Tsc1*^*fl/fl*^ (referred to hereinafter as: *p53*^*-/-*^*; Tsc1*^*-/-*^) mice were generated. Surprisingly, additional ablation of p53 did not prolong the survival of *Tsc1*^*-/-*^ mice: all *p53*^*-/-*^*; Tsc1*^*-/-*^ mice died between the age of 84 days and 237 days (*n* = 7; median survival: 101 days vs. 133 days in *Tsc1*^*-/-*^ mice, *n* = 5; Fig. 3a); however, neither significant loss of acinar cells nor death of acinar cells was observed. Instead, histological examination of *p53*^*-/-*^*; Tsc1*^*-/-*^ pancreata revealed occurrence of dysplastic acinar cells in all animals (Fig. 3b, upper panel). These cells were characterized by an increased nuclear-cytoplasmic ratio with moderate to severe nuclear atypia with coarse granular chromatin, multiple nucleoli and single atypical mitoses. Additionally, nodular hyperplasia in acinar cells (circumscribed aggregates of acinar cells with distinct tinctorial differences to the surrounding acinar cells) were observed in 4 mice (Fig. 3b, lower panel); further, a large pancreatic tumor (Fig. 3c, Additional file 1: Figure S1A) was seen in one out of seven mice. Histological examination of the tumor revealed that the tumor cells were highly proliferative and showed distinctive tumor morphology reminiscent of human ACC (Fig. 3c) with an acinar to solid growth pattern and neoplastic cells with an amphophilic to eosinophilic granular cytoplasm. Positivity of the tumor cells for secretory enzymes such as Trypsin 3 (protease, serine 3) and α-Amylase (Additional file 1: Figure S1B) underscored the acinar origin of the neoplasia.

In regard to the endocrine compartment, however, we observed a phenotype as described for *Tsc1*^*-/-*^ pancreata: no islets were seen in any of the *p53*^*-/-*^*; Tsc1*^*-/-*^ pancreata, except for one with a large ACC-like tumor. Since no difference in survival of *p53*^*-/-*^*; Tsc1*^*-/-*^ and *Tsc1*^*-/-*^ mice was found, uncontrolled diabetes mellitus seems to be the cause of death in these animals. Such changes were not seen in *p53*^*-/-*^*; Tsc1*^*-/+*^ pancreata (Additional file 1: Figure S1C).

To investigate signal changes occurring in *p53*^*-/-*^*; Tsc1*^*-/+*^ and *p53*^*-/-*^*; Tsc1*^*-/-*^ pancreata, we performed a set of phosphorylation stainings for Erk, Akt, mTOR and S6. This analysis revealed that pancreatic epithelial cells in *p53*^*-/-*^*; Tsc1*^*-/+*^ mice and tumor cells (and atypical acinar cells) in *p53*^*-/-*^*; Tsc1*^*-/-*^ mice were generally devoid of p-Akt^Ser473^ and p-Erk1/2^Thr202/Tyr204^ (Fig. 3d, e); pancreatic acinar cells of *p53*^*-/-*^*; Tsc1*^*-/+*^ mice had detectable levels of p-mTOR^Ser2448^ and p-S6^Ser235/236^; however, the staining intensities in the tumor cells (and atypical acinar cells) of *p53*^*-/-*^*; Tsc1*^*-/-*^ mice were more pronounced (Fig. 3f, g). These data suggest that hyper-activated mTOR signaling in *p53*^*-/-*^*; Tsc1*^*-/-*^ pancreata was not dependent on either the Mek/Erk nor the PI3K/Akt cascade.

In a next step, we established a set of mouse cell lines from *p53*^*-/-*^*; Tsc1*^*-/+*^ (911 and 961 cells) and *p53*^*-/-*^*; Tsc1*^*-/-*^ mice (946 F and 946 cells from ACC-like tumors). As previously reported, a set of p53-deficient cell lines underwent epithelial-to-mesenchymal transition (EMT) and exhibited fibroblast-like morphologies in vitro (Fig. 3h, upper panel) [24], except for one clone (946), which showed a stable epithelial morphology (Fig. 3h, lower panel). Cell lines that had undergone EMT (911, 961 and 946 F) expressed low levels of E-Cadherin but high levels of vimentin, compared to those without EMT features (946 cells, Fig. 3i). Neither was Tsc1 detected in the *Tsc1*-deficient cell lines (946 and 946 F) nor was p53 found in p53-deficient cells (911, 961, 946 and 946 F cells, Fig. 3i, and IHC of p53: Additional file 1: Figure S2A). To test clonogenicity and tumorigenicity of these cell lines, we performed orthotopic implantation into wild-type (WT) mice and subcutaneous injection into BALB/c nude mice. None of the tested cells lines were able to form tumors in WT mice (*n* = 24). However, tumors formed at 67 % of the sites (16/24) injected with cells with EMT features (cell lines 911, 961 and 946 F), while no tumors were found at the sites (0/3) injected with the cells without EMT features (946; BALB/c nude mice). An overview of the transplanted cells, mouse numbers and tumor formation is provided in Additional file 2: Table S1. No difference in proliferation of these cell lines in vivo was detected by quantifying the number of phospho-Histone H3 (pHH3) positive cells (Additional file 1: Figure S2B).

To further confirm the signal changes in vitro, we cultured *p53*^*-/-*^*; Tsc1*^*-/+*^ and *p53*^*-/-*^*; Tsc*^*-/-*^ cell lines under serum deprivation conditions. Only weak constitutively active Akt or Erk signaling (as demonstrated by p-Akt^Ser473^ and p-Erk1/2^Thr202/Tyr204^ staining) was observed in these cell lines, but serum treatment (20 % FBS) induced pronounced Akt and Erk activation in these cells; though to a lesser extent in the *p53*^*-/-*^*; Tsc1*^*-/-*^ cell lines. The *p53*^*-/-*^*; Tsc1*^*-/+*^ cell lines had relatively low levels of basal mTOR signaling (as demonstrated by p-mTOR^Ser2448^) which was slightly increased by serum treatment. In contrast, mTOR signaling in the *p53*^*-/-*^*; Tsc1*^*-/-*^ cells was constantly active and serum treatment had marginal additional effects (Fig. 3j).

### mTOR signaling is activated while p53 signaling is inactivated in human ACCs

**Fig. 4:**
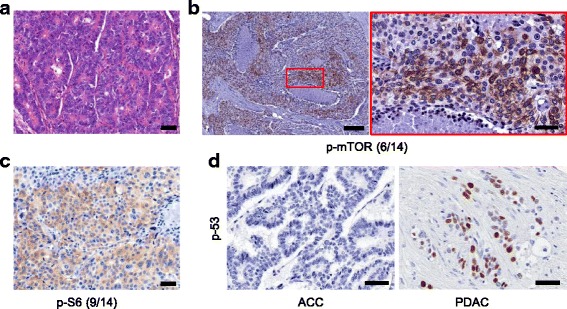
mTOR signaling is activated while p53 signaling is inactivated in human ACCs. (**a**) Representative H&E-stained sections of the histology of human ACCs; (**b**-**c**) Representative IHC pictures show distinct in vivo activation of mTOR signaling (p-mTOR^Ser2448^ and p-S6^Ser235/236^) in a subset of human ACC, but universal inactivation of p53 signalling (nuclear staining for p53), scale bar in (**b**) (*right panel*) and (**c**): 50 μm; scale bar in (**b**) (*left panel*): 200 μm; (**d**) Representative IHC pictures show inactivation of p53 signalling (nuclear staining for p53) in ACCs, but not in PDACs; scale bar: 50 μm

To investigate mTOR and p53 signaling in human ACCs, 14 ACC samples were stained (Fig. 4a) with p-mTOR^Ser2448^, p-S6^Ser235/236^ and p53 antibodies. This analysis revealed that 43 % (6/14) and 64 % (9/14) of the samples were positive for p-mTOR^Ser2448^ and p-S6^Ser235/236^, respectively (Fig. 4b, c). Notably, no nuclear p53 staining was detected in any of tested samples, indicating a universal inactivation of p53 signalling in human ACC. As a control, PDAC sections were stained with p53 antibodies (Fig. 4d).

## Discussion

The Tsc/mTOR/p53 signaling loop is a delicate feedback system by which cells are able to cope with genotoxic and nutritional stress [25]. mTOR activates p53 by either increasing its protein synthesis or its stability, which increases the GAP activity of the Tsc1-Tsc2 complex via AMPK and sestrin 1/2 [20, 21]. Hence, the Tsc1-Tsc2 complex constitutes an essential component of the signaling loop. It has been reported that hyperactive mTOR signaling - caused by loss of Tsc1 or Tsc2 - induced accumulation of p53 and apoptosis in mouse embryonic fibroblasts upon glucose starvation [23]. Consistently, we demonstrate that pancreatic acinar cells do not tolerate hyperactive mTOR signaling induced by *Tsc1* deficiency and undergo apoptosis, likely in a p53-dependent way. Additional ablation of p53 eliminated cell death, but simultaneously promoted formation of dysplastic acinar cells and induced a malignancy resembling ACC. However, degeneration of the endocrine compartment was not affected by the p53 status. These data suggest that mTOR hyperactivation-induced apoptosis of endocrine cells is not a p53-dependent process. This argument is in line with published data showing that impairment of autophagy and induction of endoplasmic reticulum (ER) stress are potentially responsible for cell death [26]. The exact mechanisms underlying such effects remain yet to be defined.

By analysing pancreas tissues of *p53*^*-/-;*^*Tsc1*^*-/-*^ mice, we observed that all samples displayed dysplastic acinar cells and four out of them showed nodular hyperplasia in the exocrine pancreas. These data suggest that the presumed precancerous lesions indeed developed in those mice without established ACC-like tumors. However, due to the general loss of islets in these mice, uncontrolled diabetes mellitus seems to have caused the early death of these animals. Thus, this “compound” phenotype in the endocrine compartment does not allow for a sufficient follow-up of ACC-like tumor development in our mouse models. Following this reasoning, the only mouse developing an ACC-like tumor had the longest survival (237 days). To resolve this issue, it is worthwhile to specifically ablate p53 and Tsc1 in pancreatic acinar cells in the adult organ using an inducible system.

Recently, Ding and co-workers reported that *Tsc1* ablation mediated by Neurogenin 3-Cre (*Neurog3*^*Cre*^*; Tsc1*^*-/-*^ mice) also induced “adenocarcinoma-like” lesions showing features of ACCs [27]. Interestingly, no massive acinar cell apoptosis/involution was described in the pancreas of *Neurog3*^*Cre*^*; Tsc1*^*-/-*^*mice*. Furthermore, ACC-like tumors developed at a relatively long latency. Of note, the *Neurog3*^*Cre*^ line is not a “usual” Cre line for studying pancreatic exocrine malignancies because it mainly mediates genetic recombination in the endocrine compartment. Lineage tracing experiments revealed that only a small percentage of acinar cells (around 0.8 %) in the adult organ were Cre-positive in the *Neurog3*^*Cre*^ line [28]. Thus, the majority of acinar cells in *Neurog3*^*Cre*^*; Tsc1*^*-/-*^ mice are spared from genetic recombination at the *Tsc1* locus. This explains why no massive acinar cell apoptosis was observed in *Neurog3*^*Cre*^*; Tsc1*^*-/-*^ mice. This lack of organ involution in the acinar cell compartment allows for long-term monitoring of tumor development in these mice. Indeed, all *Neurog3*^*Cre*^*; Tsc1*^*-/-*^ mice developed ACC-like tumors by the age of 10 months. Given the continuous DNA damage in *Tsc1*-deficient cells, it remains unknown how many of the ACC-like tumors have acquired a genetic alteration of p53 during tumor evolution. Due to the “premature” demise of the *p53*^*-/-*^*; Tsc1*^*-/-*^ mice, the real penetrance of the mTOR-p53 axis in initiating ACC-like tumors remains unclear. Nevertheless, two studies now provide consistent evidence that hyperactive mTOR is the driving force for the development of - at least a subset - of ACCs.

## Conclusions

The mTOR-p53 axis constitutes an important regulatory machinery which controls tissue homeostasis of the pancreas. Dysregulation of this axis contributes to the development of ACCs. Preclinical studies testing the effectiveness of anti-mTOR therapies in ACC mouse models are warranted.

## Methods

### Patient material and tissue collection

ACC tissues were from patients who had undergone pancreatic resections. All sample diagnoses were confirmed histologically. Samples were fixed in paraformaldehyde solution for 24 h and subsequently paraffin-embedded for histological analysis. The use of tissue for analysis was approved by the local Ethics Committee (Technical University Munich, #1926/07) and written informed consent was obtained from the patients prior to surgery (Department of Surgery, Klinikum rechts der Isar).

### Mouse lines

Mice containing a floxed allele of Tsc1 (005680) and of p53 (008462) were obtained from The Jackson Laboratory (Bar Harbor, USA). The pancreas-specific Cre recombinase line Ptf1a^Cre/+^ (also known as p48^Cre/+^) was a kind gift from Roland M. Schmid and Jens T. Siveke (Dept. of Gastroenterology, TU Munich). The wild type (WT; C57BL/6 J) and thhe BALB/c nude mice were obtained from Charles River Laboratory (Sulzfeld, Germany).

### Mouse breeding

Mouse breeding was performed and husbandry was maintained at the specific pathogen free (SPF) mouse facility at the Technical University of Munich. The compound transgenic mice were maintained on a mixed background. All mouse experiments and procedures were approved by the Institutional Animal Care and Use Committees of the Technical University of Munich. All procedures were in accordance with the Office of Laboratory Animal Welfare and the German Federal Animal Protection Laws. All mouse experiments and procedures were approved by Bavarian Government (No. 55.2-1-54-2532-42-13; 55.2-1-54-2531-33-08). Unless otherwise stated, all animals were followed up for up to 1.5 years or sacrificed for histological analysis if they showed any sign of disease.

### Primary cell isolation

Freshly dissected sterile tumor tissues were washed twice with ice-cold PBS, cut into small cubes (approximately 1 mm) and dispensed into 5 ml of complete medium containing collagenase (1.2 mg/ml). The resulting solution (mixed with tissue blocks) was incubated at 37 °C for 0.5 h. After centrifugation at 300 rpm for 5 min, the small tissue blocks were washed twice with collagenase-free medium, followed by incubation at 37 °C with medium containing collagenase for an additional 0.5 h. After passing the undigested tissue blocks through a 100 μm nylon mesh, cell suspensions were obtained. These cell suspensions were washed two times with complete medium and seeded into a 10 cm^2^ dish.

### Cell transplantation experiments

For mouse cell transplantation experiments into BALB/c nude mice, 10^6^ tumor cells were resuspended in 50 μl of PBS and were injected into the left lower and right upper flank. Tumor growth was monitored macroscopically every week. Orthotopic transplantations of mouse cells were carried out as described previously [29, 30]. Briefly, mice were anesthetized, and a left-lateral incision of the abdomen was made to visualize the tail region of the pancreas. 10^6^ tumor cells were suspended in 50 μl of PBS and were carefully injected into the pancreatic tail. The abdominal wall and skin were closed using running sutures. All mice were sacrificed for histological evaluation after 4 weeks.

### Further materials and methods

A detailed materials and methods section is provided as a Additional file 3.

### Statistical analysis

GraphPad Prism 5 Software (GraphPad, San Diego, CA, USA) was used for the statistical (survival) analysis. Statistical significance was set at *p* < 0.05.

## Additional files

Additional file 1: **Figure S1.** A, Gross pathology of one ACC-like tumor in *p53* ^*-/-*^ *; Tsc1* ^*-/-*^ mice; B, IHC demonstrates expression of α-Amylase and Trypsin 3 in tumors cells and acinar cells of *p53* ^*-/-*^ *; Tsc1* ^*-/-*^ and *p53* ^*-/-*^ *; Tsc1* ^*-/+*^ mice, respectively; scale bar: 50 μm; C, Representative H&E stained section shows normal histology of *p53* ^*-/-*^ *; Tsc1* ^*-/+*^ pancreata; scale bar: 50 μm. **Figure S2.** A, Representative IHC pictures demonstrate the knockout status of p53 in *p53* ^*-/-*^ *; Tsc1* ^*-/-*^ and *p53* ^*-/-*^ *; Tsc1* ^*-/+*^ pancreata, respectively; scale bar: 100 μm; B, Quantification of proliferating cells (marked by p-HH3 staining) in tumors that developed after implantation of *p53* ^*-/-*^ *; Tsc1* ^*-/-*^ (946 F) and *p53* ^*-/-*^ *; Tsc1* ^*-/+*^ (911 and 961) cell lines into BALB/c nude mice shows no differences; n.s.: no significant change tested by one-way ANOVA. (PPT 38925 kb)

Additional file 2: Table S1. Tumor formation by *p53* ^*-/-*^ *; Tsc1* ^*-/-*^ or *p53* ^*-/-*^ *; Tsc1* ^*-/+*^ cells in wild-type (WT) and BALB/c nude mice. (DOC 28 kb)

Additional file 3: **Supplementary Materials and Methods.** (DOC 59 kb)

## Abbreviations

ACC: acinar cell carcinoma
mTOR: mammalian target of rapamycin
PI3K: phosphatidylinositol-4, 5-bisphosphate 3-kinase
Tsc: Tuberous sclerosis complex
Tsc1: hamartin
