# Supplementary material for: Pancreas-specific activation of mTOR and loss of p53 induce tumors reminiscent of acinar cell carcinoma
Source: Mol Cancer. 2015 Dec 18;14:212. doi: 10.1186/s12943-015-0483-1 (PMC4683950; doi:10.1186/s12943-015-0483-1)
Supplement: Supplementary file 2 — Tumor formation by p53 -/- ; Tsc1 -/- or p53 -/- ; Tsc1 -/+ cells in wild-type (WT) and BALB/c nude mice. (DOC 28 kb) [file 12943_2015_483_MOESM2_ESM.doc]

**Table S1**

Tumor formation by *p53^-/-^; Tsc1^-/-^* or *p53^-/-^; Tsc1^-/+^* cells in wild-type (WT) and BALB/c nude mice

| Cell lines | Genotype | Primary lesions | WT mice | | BALB/c nude mice | |
| --- | --- | --- | --- | --- | --- | --- |
|  |  |  | Tumor/Total | Rate (%) | Tumor/Total | Rate (%) |
| 911 | *p53^-/-^; Tsc1^-/+^* | Normal | 0/4 | 0% | 4/6 | 67% |
| 961 | *p53^-/-^; Tsc1^-/+^* | Normal | 0/4 | 0% | 5/6 | 83% |
| 946 | *p53^-/-^; Tsc1^-/-^* | ACC-like | 0/8 | 0% | 0/3 | 0% |
| 946F | *p53^-/-^; Tsc1^-/-^* | ACC-like | 0/8 | 0% | 7/9 | 78% |
