# Supplementary material for: Pancreas-specific activation of mTOR and loss of p53 induce tumors reminiscent of acinar cell carcinoma
Source: Mol Cancer. 2015 Dec 18;14:212. doi: 10.1186/s12943-015-0483-1 (PMC4683950; doi:10.1186/s12943-015-0483-1)
Supplement: Supplementary file 3 — Supplementary Materials and Methods. (DOC 59 kb) [file 12943_2015_483_MOESM3_ESM.doc]

**Supplementary Materials and Methods**

**Mouse breeding**

Mouse breeding was performed and husbandry was maintained at the specific pathogen free (SPF) mouse facility at the TU Munich. The compound transgenic mice were maintained on a mixed genetic background. All mouse experiments and procedures were approved by the Institutional Animal Care and Use Committees of the Technical University of Munich and the Upper Bavaria Office of Laboratory Animal Welfare (Regierung von Oberbayern). All procedures were in accordance with the German Federal Animal Protection Laws.

**Mouse cell lines**

The primary mouse cell lines 911, 961 were isolated from transgenic *p53^-/-^; Tsc1^-/+^* mice, respectively. 946 and 946F cell lines were two different clones isolated from the same *p53^-/-^; Tsc1^-/-^* mice with ACC-like tumor.

**List of antibodies**

**Primary antibodies**

| Antibody name | Catalog number | Application*  (Reactivity**) | Producer |
| --- | --- | --- | --- |
| Rabbit Anti-p-Akt^Ser473^ mAb^#^ | 4060 | WB; IHC (M) | Cell Signaling Technology |
| Rabbit Anti-Akt Ab^#^ | 9272 | WB (M) | Cell Signaling Technology |
| Rabbit Anti-p-Erk1/2^Thr202/Tyr204^ Ab^#^ | 9101 | WB; IHC (M) | Cell Signaling Technology |
| Rabbit Anti-Erk1/2 Ab^#^ | 9102 | WB (M) | Cell Signaling Technology |
| Rabbit Anti-p-mTOR^ser2448^ mAb^#^ | 5536 | WB (M) | Cell Signaling Technology |
| Rabbit Anti-p-mTOR^Ser2448^ mAb^#^ | 2976 | IHC (H, M) | Cell Signaling Technology |
| Rabbit Anti-Pten mAb^#^ | 9188 | WB; IHC (M) | Cell Signaling Technology |
| Rabbit Anti-mTOR mAb^#^ | 2983 | WB; IHC (M) | Cell Signaling Technology |
| Rabbit Anti-p-S6^Ser235/236^ Ab^#^ | 2211 | WB; IHC (M) | Cell Signaling Technology |
| Rabbit Anti-p-Histone H3^Ser10^ Ab^#^ | 9701 | IHC (M) | Cell Signaling Technology |
| Rabbit Anti-Glucagon Ab^#^ | 2760 | IHC; IF  (M) | Cell Signaling Technology |
| Rabbit Anti-α-Amylase mAb^#^ | 3796 | WB; IHC; IF  (M) | Cell Signaling Technology |
| Rabbit Anti-E-Cadherin mAb^#^ | 3195 | WB; IHC  (M) | Cell Signaling Technology |
| Rabbit Anti-Tsc1 mAb^#^ | 6935 | WB (M) | Cell Signaling Technology |
| Mouse Anti-Insulin mAb^#^ | ab8304 | IHC, IF (M) | Abcam (Cambridge, UK) |
| Rabbit Anti-GAPDH Ab^#^ | sc-25778 | WB (H, M) | Santa Cruz biotechnology (Heidelberg, Germany) |
| Rabbit Anti-p53 Ab^#^ | M362929-2 | IHC (H) | Dako Deutschland GmbH (Hamburg, Germany) |
| Mouse Anti-Vimentin mAb^#^ | ab8978 | WB (M) | Abcam (Cambridge, UK) |
| Mouse Anti-β actin Ab^#^ | sc-69879 | WB (H, M) | Santa Cruz biotechnology |
| Rat Anti-Krt19 Ab# | TROMA-Ⅲ | IHC (M) | Developmental Studies Hybridoma Bank (Iowa, USA) |

Secondary antibodies

| Antibody name | Catalog number | Application* | Producer |
| --- | --- | --- | --- |
| Rabbit HRP (horseradish peroxidase)- labelled Anti-Rat  IgG Ab^#^ | P0450 | IHC | Dako Deutschland GmbH (Hamburg, Germany) |
| Goat HRP-Labelled Polymer Anti-Mouse Ab^#^ | K4001 | IHC | Dako Deutschland GmbH |
| Goat HRP-Labelled Polymer Anti-Rabbit Ab^#^ | K4003 | IHC | Dako Deutschland GmbH |
| Goat Alexa Fluor 488 Anti-Mouse IgG Ab^#^ | 115-546-062 | IF | Dianova (Hamburg, Germany) |
| Chicken Alexa Fluor 594 Anti-Rabbit IgG Ab^#^ | A-21442 | IF | Invitrogen  (Carlsbad, CA, USA) |
| Goat Alexa Fluor 594  Anti-Rat IgG Ab^#^ | A-11007 | IF | Invitrogen |
| Sheep HRP-labelled Anti-Mouse IgG Ab^#^ | NA931 | WB | GE Healthcare  (Little Chalfont, UK) |
| Donkey HRP-labelled Anti-Rabbit IgG Ab^#^ | NA934 | WB | GE Healthcare |
| Donkey HRP-labelled Anti-Goat IgG Ab^#^ | sc-2020 | WB | Santa Cruz biotechnology |

*Application key: WB = western-blot; IHC = Immunohistochemistry; IF = Immunofluorescence; ^**^Reactivity key: H = human; M = mouse; ^#^Ab: antibody

**Immunohistochemistry analysis**

Immunohistochemistry was performed using the Dako Envision System (Dako Cytomation GmbH, Hamburg, Germany). Consecutive paraffin-embedded tissue sections (3–5 mm thick) were deparaffinized and rehydrated using routine methods. Antigen retrieval was performed by pretreat­ment of the slides in citrate buffer (pH 6.0; 10mM Citric Acid, 0.05% Tween 20) in a microwave oven for 10 minutes. Endogenous peroxidase activity was quenched by incubation in deionized water containing 3% hydrogen peroxide at room temperature for 10 minutes. After blocking of nonspecific reactivity with TBS (pH 7.4; 0.1M Tris Base, 1.4M NaCl) containing 3% BSA or goat serum, sections were incubated with the respective antibody at 4˚C overnight followed by incubation with horseradish peroxidase-linked goat anti-rabbit or mouse antibodies, followed by a color-reaction with diamino­benzidine and counterstaining with Mayer’s hematoxylin.

**Immunofluorescence**

Paraffin-embedded tissue sections (3 µm thick) were deparaffinized and rehydrated using routine methods, were permeabilized with 0.1% Triton X-100, and were incubated with primary antibodies overnight at 4°C. After washing, secondary antibodies and 4,6-diamidino-2-phenylindole (DAPI) were used accordingly.

**Cell culture and treatment**

Human or mouse cell lines were cultured in 10 cm dishes either in DMEM or RPMI-1640 cell culture medium supplemented with 10% fetal bovine serum (FBS), 100 u/ml penicillin and 100 µg/ml streptomycin at 37°, 5% CO2.

**Immunoblot analysis**

*Protein extraction from cells*

Cells were washed twice with ice-cold PBS (pH 7.4; 0.01M PBS). After addition of ice-cold modified RIPA buffer/or cell lysis buffer containing 20 mM Tris-HCl (pH 7.5), 150 mM NaCl, 1 mM Na_2_EDTA, 1 mM EGTA, 1% NP-40, 1% sodium deoxycholate, 2.5 mM sodium pyrophosphate, 1 mM β-glycerolphosphate, 1mM NaVO_4_, 1 µg/ml leupeptin and 1 mM PMSF, cells were immediately homogenized by passing them through a G27 syringe needle 10 times. The crude homogenate was then centrifuged at 14,000 g in a pre-cooled centrifuge for 15 minutes. The supernatant was immediately transferred to fresh tubes and was aliquoted. The concentration of the extracted protein was determined using the BCA Protein Assay kit (Pierce, Thermo Scientific, USA) following the manufacturer’s instructions. The sample aliquots were stored at -20ºC or were used for Western blotting analysis immediately.

*Western blotting*

20 to 80 µg of the total protein were loaded on 4~12% polyacrylamide gels and were transferred to PVDF membranes. Membranes were blocked in 20 ml Tween-20 (0.05%)-TBS (pH 7.4; 0.1M Tris Base, 1.4M NaCl) containing 3% or 5% milk for 1 hour and were incubated with the respective primary antibody overnight at 4˚C. Membranes were washed 3 times with 0.05% Tween-20-TBS and were incubated with a horseradish peroxidase (HRP)-conjugated secondary anti­body (1:3000) for 1h at room temperature. Signals were detected using the enhanced chemiluminescence system (ECL, Amersham Life Science Ltd., Bucks, UK). Films were scanned with a CanoScan 9900F scanner (Canon, Tokyo, Japan).
